# Genetic Diversity Analysis of Genotype 2 Porcine Reproductive and Respiratory Syndrome Viruses Emerging in Recent Years in China

**DOI:** 10.1155/2014/748068

**Published:** 2014-02-25

**Authors:** Lei Zhou, Xiaorong Yang, Yuan Tian, Shuoyan Yin, Gang Geng, Xinna Ge, Xin Guo, Hanchun Yang

**Affiliations:** Key Laboratory of Animal Epidemiology and Zoonosis of Ministry of Agriculture, College of Veterinary Medicine and State Key Laboratory of Agrobiotechnology, China Agricultural University, No. 2 Yuanmingyuan West Road, Haidian District, Beijing 100193, China

## Abstract

Porcine reproductive and respiratory syndrome virus (PRRSV) is characterized by its extensive genetic diversity. Here we analyzed 101 sequences of NSP2 hypervariable region, 123 ORF3 sequences, and 118 ORF5 sequences from 128 PRRSV-positive clinical samples collected in different areas of China during 2008–early 2012. The results indicated that the amino acid identities of the three genes among these sequences were 87.6%–100%, 92.5%–100%, and 77%–100%, respectively. Meanwhile, 4 novel patterns of deletion and insertion in NSP2 region or GP5 were first found. The phylogenetic analysis on these 3 genes revealed that the Chinese PRRSV strains could be divided into three subgroups; majority of genes analyzed here were clustered in subgroup 3 with multiple branches; the strains with 30-aa deletion in NSP2-coding region were still the dominant virus in the field. Further phylogenetic analysis on four obtained complete genomic sequences showed that they were clustered into different branches with the Chinese corresponding representative strains. Our analyses suggest that the genetic diversity of genotype 2 PRRSV in the field displays a tendency of increasing in recent years in China, and the 30-aa deletion in NSP2-coding region should be no longer defined as the molecular marker of the Chinese HP-PRRSV.

## 1. Introduction

Porcine reproductive and respiratory syndrome (PRRS) characterized as reproductive failure in sow and respiratory disorder in all-age pigs [1] is regarded as one of the major concerns for disease controlling in pig farms [2–5]. The first outbreak of PRRS in Western Europe and North America was almost concurrently documented during the late 1980s and early 1990s [6, 7]. Within the succeeding years, PRRS was an endemic disease in North America, Europe, and Asia [7–11]. Since then, PRRS has become the most economically devastating disease for global pig industry [4, 5].

The causal agent, porcine reproductive and respiratory syndrome virus (PRRSV), is classified into the order Nidovirales, family Arteriviridae, together with equine arteritis virus (EAV), lactate dehydrogenase-elevating virus (LDV), and simian hemorrhagic fever virus (SHFV) [12, 13]. According to the genetic diversity, PRRSV can be divided into two genotypes: type 1 (European) PRRSV with prototype Lelystad and type 2 (North American) PRRSV with prototype VR-2332. Although the two types of PRRSV can cause similar syndrome to the infected pigs, they share only 55%–70% nucleotide and 50%–80% amino acid similarity in their various genes [14]. The single positive-strand RNA genome of PRRSV is approximately 15 kb in length, encoding at least 10 open reading frames (ORF) [15–18]. The ORF1a and ORF1b encode replication-related polymerase proteins, which can be autoproteolytically cleaved into at least 13 nonstructural proteins (NSP) [19–22]. And the rest of ORFs 2 to 7 encode viral structural proteins [15, 17, 23, 24]. Among them, the largest nonstructural protein gene—NSP2, ORF3 encoding minor glycosylated structural protein—GP3, and ORF5 that encodes major envelope protein—GP5 are often selected for variation investigation and phylogenetic analyses for their genetic diversities [25, 26]. The genetically extensive variation with genetic/antigenic diverse strains in the field is regarded as an important reason for vaccination failure and occasional outbreaks of more severe forms of PRRS [21, 26].

Since the first outbreak of PRRS in China was documented at the end of 1995 [27], this disease has been accompanying the Chinese swine industry [28]. Considering China has the largest number of pig farms with diversity of size and different levels of biosecurity control and management, the economical cost caused by PRRS in China should be higher than that in the United States, which was estimated to be $664 million per year [4, 29]. Especially in 2006, a large-scale outbreak caused by the highly pathogenic PRRSV (HP-PRRSV) was characterized by prolonged high fiver, rubefaction on the skin, and increased morbidity and mortality in all ages of pigs, resulting in unprecedented damage to the Chinese swine industry [25, 30, 31]. The phylogenetic analyses have indicated that the causative pathogen HP-PRRSV was evolved by a gradual variation and accumulation progress of genome changes from the early Chinese domestic strain [25, 26]. In the following years, the HP-PRRSV has been becoming the dominant strains in the field [25]. In the year 2011, the Chinese HP-PRRSV-derived commercial vaccines, which were attenuated by serial passaging on the MARC-145 cells, were approved to put on the domestic market. In the same year, the European PRRSV isolates were first reported in China [32]. Considering the risk of potential reversion to virulence and recombination, the two events increased the complexity of PRRSV epidemic situation in China, which will attract more attention on the molecular epidemiology analysis.

In this study we phylogenetically analyzed the PRRSV NSP2 hypervariable (HV) region and ORF3 and ORF5 genes, which were directly amplified from the clinical samples collected from various pig farms around the pig-producing areas of China, during the period from 2008 to early 2012. Simultaneously, we described the complete genomic sequences of four new Chinese PRRSV isolates including one strain from Tibet mini-pig and three sharing novel characteristic genetic variations and compared their genetic characterization with previous strains. Finally a phylogenetic tree based on the full-length genomic sequence is conducted in order to analyze the evolutionary relationship of these strains.

## 2. Materials and Methods 

### 2.1. Sample Collection and Geographic Distribution

During the period from 2008 to early 2012, 128 clinical samples, including lung, brain, spleen, lymph node, and sera, which were positive for PRRSV by conventional laboratory detection and diagnosis, were collected from pig farms distributed in 18 regions of China. These samples were further used for PRRSV isolation or NSP2 HV region and ORF3 and ORF5 genes amplification and sequencing.

### 2.2. RNA Extraction and RT-PCR Amplification and Sequencing

Total RNA was extracted from 250 *μ*L of tissue homogenates or serum by using TRIzol LS reagent (Invitrogen Corporation, Auckland, NY, USA). Then reverse transcription was performed by using M-MLV reverse transcriptase (Promega, Madison, WI, USA) and specific antisense primers (Table 1). Resulting cDNA was amplified by using PrimeSTAR HS DNA polymerase (TaKaRa Biotechnology Co., Dalian, China) in the following process: 34 cycles of denaturation at 98°C for 12 s, annealing at 56°C for 10 s, and extension at 72°C for 1 min/kb. The PCR products were examined by gel electrophoresis and purified by using Agarose Gel DNA Extraction Kit (BioDev Co., Beijing, China) and then subjected to BGI (Beijing, China) for sequencing.

### 2.3. Cells and Virus

MARC-145 cells were grown at 37°C in Dulbecco's minimum essential medium (DMEM) supplemented with 10% fetal bovine serum (FBS) and antibiotics. The pulmonary alveolar macrophage (PAM) cells were prepared as described previously [33] and maintained in 10% FBS 1640 medium. Serum or supernatant of tissue homogenates from PRRSV-positive samples were used to inoculate the MARC-145 cells or PAM for PRRSV isolation.

### 2.4. Full-Length Genomic Sequencing of PRRSV Isolates

Fourteen pairs of primers for genotype 2 PRRSV (Table 1), covering the full-length genomes, were designed, based on JXwn06 (Accession number EF641008). Each fragment of the isolates was amplified and cloned into pEASY-Blunt vector (Transgen Tech Co, Beijing, China) as described previously [34]. The 5′ and 3′ ends region was amplified using 5′ and 3′ full RACE kit (TaKaRa, Dalian, China) according to the manufacturer's instructions. The PCR products or plasmid with cloned PRRSV fragments was subjected to BGI (Beijing, China) for sequencing.

### 2.5. Sequence Alignment and Phylogenetic Analysis

The nucleotide and deduced amino acid sequences were aligned by ClustalW in software Lasergene (DNASTAR Inc., Madison, WI, USA) to determine sequence homology. And phylogenetic and molecular evolutionary analyses were conducted using MEGA version 5 (Tamura, Peterson, Peterson, Stecher, Nei, and Kumar 2011), along with multiple sequences of representative PRRSV available in GenBank from various countries and areas (Supplementary Table S1, see Table S1 in Supplementary Material available online at http://dx.doi.org/10.1155/2014/748068).

## 3. Results

### 3.1. Number of NSP2 HV Fragment and ORF3 and ORF5 Genes Amplified from PRRSV-Positive Clinical Samples

The fragments of NSP2 HV region and ORF3 and ORF5 genes amplified from PRRSV-positive samples were sequenced. The results showed that totally 101 NSP2, 123 ORF3, and 118 ORF5 sequences were successfully obtained from 128 PRRSV-positive samples collected during the period from 2008 to early 2012 (Table 2).

### 3.2. Sequence Alignment and Phylogenetic Analysis of NSP2 HV Region

The amplified NSP2 HV region exhibited various sizes in length (Table 2). Nucleotide and deduced amino acid sequences analysis revealed that 86 out of 101 NSP2 HV region sequences had the same length of 1014 nucleotide (nt), containing the same 30-aa deletion at aa 482 and aa 533–561 as JXwn06 and other HP-PRRSV strains, compared with the type 2 prototype VR-2332 and the Chinese early strains. The LN1101 and GZ1101 showed two novel deletion patterns in their NSP2 regions, whose nucleotide sequences length was 1050 nt and 1095 nt, respectively. The other 13 NSP2 sequences were 1104 nt in length, same as those of VR-2332.

Pairwise comparisons showed that those 86 sequences with 30-aa deletions in NSP2 shared 87.6%–100% amino acid similarity with each other. And their amino acid similarities with JXwn06 ranged from 91.7% to 99.4%, as well as 66.6% to 69.5% compared with VR-2332. Majority of the sequences without deletion shared high homology with HB-1(sh)/2002, showing the amino acid similarity of 98.1%–99.2%. Meanwhile, the JL1101 and GZ1101 displayed the highest homology with VR-2332, with amino acid similarities of 99.2% and 96.7%, respectively.

**Figure 1:**
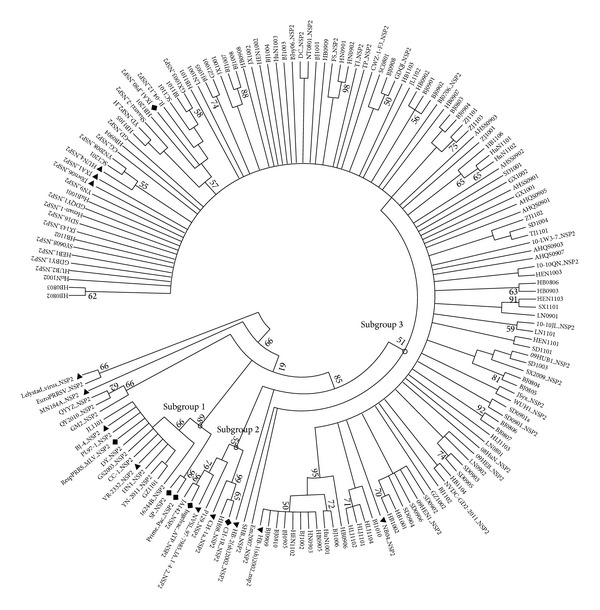
Phylogenetic tree based on the deduced amino acid sequence of NSP2 HV region. The bootstrap consensus tree is shown. The sequence downloaded from GenBank had a suffix “NSP2”. The representative strains were labeled with “black triangle” and the vaccine strains were labeled with “black diamond.” The bootstrap values were shown close to the branches.

To further gain a better understanding of the genetic relationship, the phylogenetic analysis based on deduced amino acid sequence of NSP2 HV region was conducted by using the 101 NSP2 sequences obtained in this study together with downloaded representative sequences (Supplementary Table S1). The phylogenetic tree revealed that all 101 NSP2 sequences belonged to genotype 2 of PRRSV and all Chinese PRRSV strains could be classified into three main subgroups (Figure 1). The JL1101 and GZ1101 were located in subgroup 1 with the representative strains VR-2332, BJ-4, and RespPRRS MLV, the other 99 were clustered into the subgroup 3 with multiple branches, together with the representative strains HB-1(sh)/2002, JXwn06, JXA1, and JXA1 P80. No strains in this study were clustered into subgroup 2 with representative strain CH-1a, the earliest Chinese strain. This means that the genetic diversity of NSP2 still existed and the strains with 30-aa deletion in NSP2-coding region remain to be the dominant viruses in the field. Compared with the data from 2006 to 2007, the percentage of NSP2-deleted strains increased [25]. However, these subgroups did not appear to be associated with epidemiological features based on geography or date.

Interestingly, a minor branch with JXA1 P80, the HP-PRRSV JXA1 derived vaccine strain, was observed in the NSP2 phylogenetic tree. Four strains HB1105, HB1201, SC1101, and BJ1101, collected later than the year 2011 when the JXA1-derived vaccine was launched commercially, were also clustered in this branch, whereas the parental strain JXA1 was out of this branch, suggesting that there is the possibility that the four strains directly derived from the vaccine strain JAX1 P80. However few earlier strains were also clustered into this minor branch. Even though the analysis from this study does not fully reflect that a great number of emergence of PRRSV were due to the use of HP-PRRSV-derived MLV, the potential risk of the reversion of MLV to virulent strains, and the recombination between the vaccine virus and field viruses are worthy to pay more attention to in the future [35].

### 3.3. Sequence Alignment and Phylogenetic Analysis of ORF3 Gene

All the obtained ORF3 genes in this study had the same size of 725 nt. The sequences alignments indicated that they shared 92.5%–100% amino acid similarity with each other and 89.4%–95.3% amino acid similarity with JXwn06, as well as 80.7%–85.0% with VR-2332. The regions residues 33–46, 120–133, and 162–198 were conserved among these strains; otherwise, majority of amino acid substitutions were located in two hypervariable regions, the residues 58–71 and 216–226. Especially, 63 out of 123 contained the I66-T66 mutation, comparing with those in JXwn06 and VR-2332.

**Figure 2:**
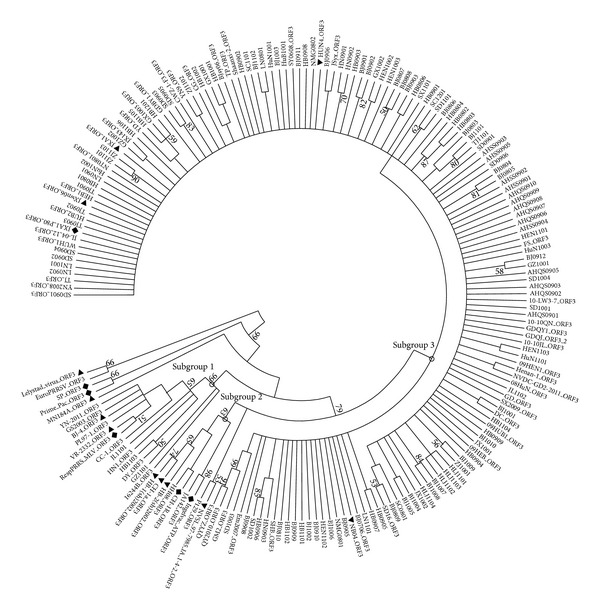
Phylogenetic tree based on the deduced amino acid sequence of ORF3. The bootstrap consensus tree is shown. The sequence downloaded from GenBank had a suffix “ORF3.” The representative strains were labeled with “black triangle” and the vaccine strains were labeled with “black diamond.” The bootstrap values were shown close to the branches.

The phylogenetic analysis of deduced amino acid sequences of ORF3 indicated that all Chinese genotype 2 strains were distributed into three subgroups (Figure 2). Three genes JL1101, HB1103, and GZ1101 were clustered into subgroup 1 with the representative strains VR-2332 and BJ-4, and no strains in this study were clustered into subgroup 2 with the representative strains CH-1a, HB-1(sh)/2002, and HB-2(sh)/2002. All the other strains were clustered into subgroup 3, which contained most Chinese strains collected later than 2004.

### 3.4. Sequence Alignment and Phylogenetic Analysis of ORF5 Gene

Except for the GZ1101 which had one amino acid deletion at the position aa 34 in ORF5-coding region, the other 117 genes had the same size of 603 nt as that of VR-2332. Sequences alignments showed that the amino acid similarity among the 117 ORF5 genes ranged from 77.0% to 100%, and they shared 78%–99% amino acid similarity with VR-2332, as well as 86.5%–99% with JXwn06. Similar as previous report, the residue 3–39, the putative signal sequence was the most variable region, whereas, the regions 40–57, 67–90, 107–120, 138–160, and 165–184 were relatively conserved [25]. However a novel substitution E170-G170, which was conserved in the Chinese strains collected during the period from 2006 to 2007, was observed in recent strains.

**Figure 3:**
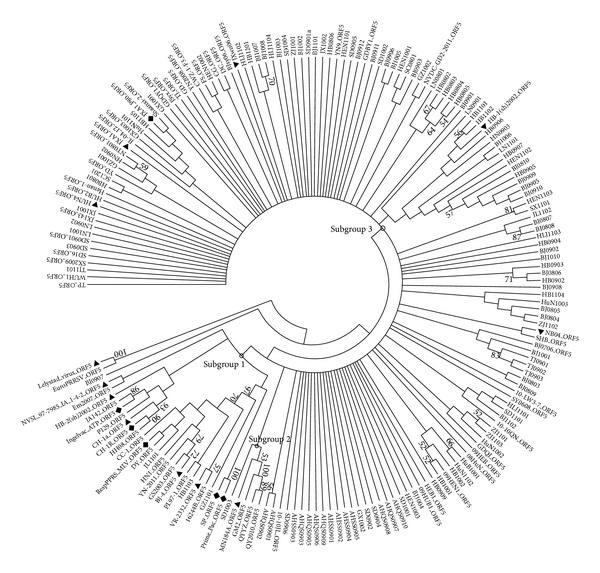
Phylogenetic tree based on the deduced amino acid sequence of ORF5.The bootstrap consensus tree is shown. The sequence downloaded from GenBank had a suffix “ORF5.” The representative strains were labeled with “black triangle” and the vaccine strains were labeled with “black diamond.” The bootstrap values were shown close to the branches.

The phylogenetic tree conducted by using the deduced amino acid sequences of ORF5 showed that the Chinese strains of genotype 2 PRRSV could be divided into 3 different subgroups (Figure 3). Three strains JL1101, HB1103, and GZ1101 were in subgroup 1 with the representative strains VR-2332, BJ-4, and CH-1a, and the SD1003 was the only strain clustered in subgroup 2 with the representative strain MN184A; all other 115 strains were clustered into subgroup 3 with multiple branches, which were completely composed of Chinese strains with the representative JXwn06 and HB-1(sh)/2002. Similar to the NSP2 phylogenetic tree, a minor branch with the JXA1 P80 contained the strains collected both earlier and later than 2011.

### 3.5. Full-Length Genomic Analysis of 4 New PRRSV Isolates

Three strains, SD0901, LN1101, and GZ1101, with characteristic deletion or insertion in NSP2 or ORF5 genes, and another strain BJ1102 were successfully isolated from the clinical samples using MARC-145 cells or PAMs. The four strains were subjected to full-length genomic sequencing after plaque purification of three rounds. The SD0901 (GenBank Accession number NJ256115) and BJ1102 (GenBank Accession number KF751237) shared same size of complete genome with 15,320 nt in length, excluding the ploy (A) tails. The genome sizes of LN1101 (GenBank Accession number KF751238) and GZ1101 were 15,356 nt and 15,404 nt, respectively. The BJ1102 was isolated from clinical samples of Tibet mini-pig with acute PRRS symptom in a pig farm where HP-PRRSV-derived vaccine was used before importing Tibet mini-pig.

**Figure 4:**
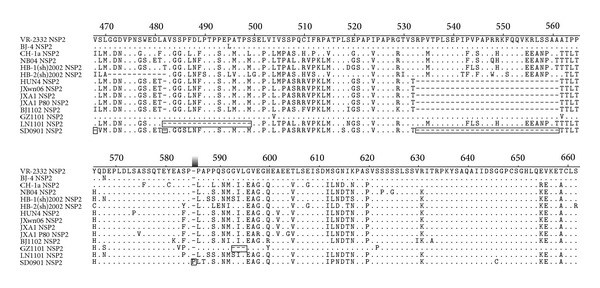
The alignment of NSP2 amino acid sequence of PRRSV. A multiple alignment of PRRSV NSP2 amino acid sequences was performed by ClustalW. The sequence of VR-2332 is shown on the top; the residues conserved with it are hidden. The deleted or inserted residues are labeled with box.

**Figure 5:**
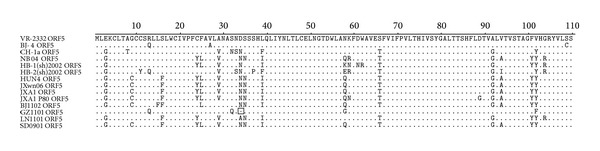
The alignment of PRRSV ORF5 amino acid sequence. Multiple alignments of PRRSV ORF5 amino acid sequences were performed by ClustalW. The sequence of VR-2332 is shown on the top; the residues conserved with it are hidden. The deleted residues are labeled with box.

Sequence alignments indicated that the 5′UTR of the four strains shared nucleotide identities of 91.0%–100% with the representative genotype 2 PRRSV strains. A nucleotide “A” insertion at the position nt 75 of GZ1101 5′UTR was first observed in this study. It was shown that major variations were located in NSP2-coding region including 3-aa deletion at the position aa 593–595 in GZ1101, 18-aa deletion at the position aa 482–499 in LN1101, 30-aa deletion at the positions aa 482 and aa 533–561 in BJ1102, and 31-aa deletion at the positions aa 468, aa 482, and aa 533–561 and an amino acid “P” insertion between aa 585 and aa 586 in SD0901 (Figure 4). In addition, a new deletion at the position aa 34 of GP5 was found in GZ1101 (Figure 5). The individual homology analysis of the other genes was also summarized in Supplementary Tables S2–S5.

**Figure 6:**
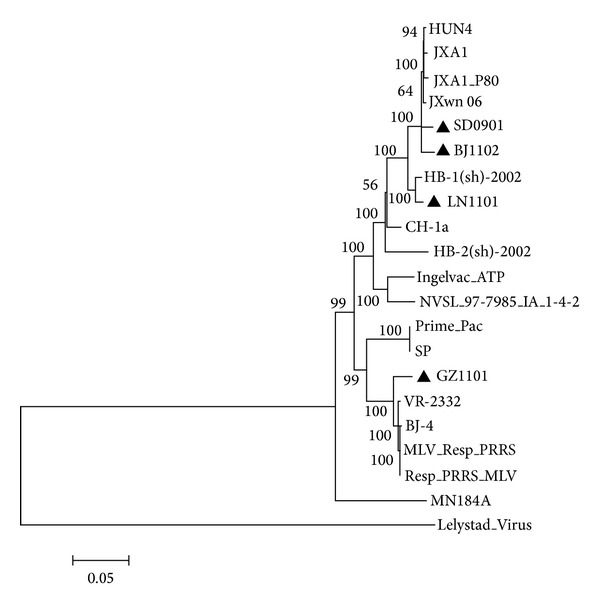
Phylogenetic tree based on full-length genomic sequence of PRRSV. The strains isolated in this study were labeled with “black triangle.” The bootstrap values were shown close to the branches. The numbers below the scale bar indicate amino acid substitution (100x).

To further classify the evolutionary relationship of these 4 isolates, the phylogenetic tree was conducted based on their full-length genomic sequence, together with both genotype 1 and genotype 2 representative strains. It was shown that the SD0901 and BJ1102 were clustered in the subgroup of Chinese HP-PRRSV and HP-PRRSV-derived vaccine virus, sharing high identity 98.7% and 98.4% with JXwn06, respectively; in addition, the LN1101 was the neighbor of HB-1(sh)/2002 in the same minor branch, which share 98.8% identity with each other. The GZ1101 was close to the minor branch with prototype VR-2332 and BJ-4 (Figure 6). The four strains exhibited 88.3%–97.8% nucleotide identity with each other. The findings suggest that various PRRSV strains from different clusters simultaneously circulate and spread in pig farms in China.

## 4. Discussion

PRRSV is characterized of its extensive genetic/antigenic variation in the field [36]. Low replication fidelity of RNA polymerase, abundance of quasispecies, RNA recombination, and immune pressure selection are regarded as the mechanisms of generating viral heterogeneity and diversity which promotes the evolution of PRRSV [37–39]. The emergence and reemergence of acute form PRRS is often influenced by the genetics of PRRSV [36]. Since the PRRS outbreak in China was first documented in 1995; this virus is always accompanied with the Chinese pig industry [27]. In 2006, an unparalleled, large-scale, atypical PRRS outbreak was reported in China [25, 30, 31]. In the following 1-2 years, the HP-PRRSV with 30-aa deletion in NSP2-coding region rapidly became the dominant in the field, meanwhile the classical and low-pathogenic strains could also be isolated from pig farms [25]. In 2011, the HP-PRRSV-derived MLV was licensed and widely used afterward in the field. This situation might greatly increase the immune selective pressure in pig herds to accelerate the variation and evolution of PRRSV [39]. Meanwhile, the European genotype 1 PRRSV strains also emerged in China [32], resulting in the complexity of PRRS. Therefore it is meaningful to continually survey the diversity of PRRSV and analyze the phylogenetic relationship and evolutionary process of field strains.

In this study we amplified and gained 101 NSP2 HV region sequences from 128 PRRSV-positive clinical samples. Out of them, 86 had the same 30-aa deletion in NSP2-coding region as that of JXwn06 and other early HP-PRRSV strains. The 86 new sequences shared 87.6%–100% amino acid similarity with each other, as well as 66.6%–69.5% with VR-2332, which were both lower than previous corresponding data, 93.4%–99.8% and 77.1%–77.8%, we obtained in 2006-2007 [25]. Meanwhile, 3 novel patterns of deletion or insertion in NSP2-coding region were first found in this study. These results suggest that the diversity of PRRSV NSP2 region has expanded from 2006-2007 to 2008–2012. The phylogenetic analysis on amino acid sequence of NSP2 indicated that all new strains in this study were clustered into 2 out of 3 subgroups: 2 strains in subgroup 1 with the representative strains VR-2332, BJ-4, and RespPRRS MLV and the other 99 in the subgroup 3 with the representative strains HB-1(sh)/2002, JXwn06, JXA1, and JXA1 P80, suggesting that the strains with 30-aa deletion in NSP2-coding region are still prevailing in the field. Among them, the BJ1102 with low pathogenicity (data not shown), which was closely related with vaccine virus, was clustered together with HP-PRRSV-derived vaccine virus in the same branch. As more and more low pathogenic strains have been found to have 30-aa deletion in NSP2-coding region, this deletion will no longer be defined as the molecular marker of HP-PRRSV.

The ORF3 sequences alignment showed that the residues within 3 regions including 33–46, 120–133, and 162–198 were relatively conserved among the strains in this study, while the nt 58–71 and 216–226 of ORF 3 gene were hypervariable regions. More than 50% strains contained the I66-T66 mutation, which was located at the identified epitope in GP3 [40–43]. A previous clue suggested that the residue substitution at this position may be related with inducing neutralizing antibody [40]. Whether this mutation is associated with the immune pressure selection or immune invasion still needs further investigation.

Even if the ORF5 is the highest variable region of PRRSV structural proteins, the deletion in this region is little recognized. In this study one amino acid deletion at the position aa 34 of GP5 was first found in GZ1101, and the sequence alignment showed that this strain had higher homology with VR-2332 and RespPRRS MLV, implying that the virus might be evolved from the vaccine virus. The lowest amino acid similarity of ORF5 among these strains was 77.0%, which was lower than the data (84.1%) in our previous research, supporting that the diversity of strains has increased since 2008. The phylogenetic tree based on deduced amino acid sequence of ORF5 showed that the Chinese PRRSV strains could be clustered into 3 different subgroups. Compared with Shi Mang's phylogenetic result based on more than 8,000 sequences, subgroup 1 was composed of representative strains located in lineage 8 (VR-2332 and CH-1a), lineage 5.1 (VR-2332 and BJ-4), and lineage 7 (SP and prime Pac), subgroup 2 contained representative strains MN184a from lineage 1, and the other HP-PRRSV in subgroup 3 was late clustered into lineage 8 in Yanyan Ni's modified phylogenetic tree, even if the information of Chinese HP-PRRSV had not been included in Shi's analysis [44, 45].

Because of having novel genetic characterization or being isolated from special host Tibet mini-pigs, four strains, SD0901, LN1101, GZ1101, and BJ1102, in our study were subjected to full-length genomic sequencing in order to better understand their characterizations of whole genome. Comparative analysis showed that their complete genome sequence homology ranged from 88.3% to 97.8%, and they were clustered into different branches of genotype 2 PRRSV, further indicating that PRRSV strains with genetic diversity simultaneously exist in the field in China.

In this study, the molecular sequence data of PRRSV was utilized to characterize the epidemiology and evolutionary process in phylogenetic analysis, expecting that it could provide an important clue for modification of diagnosis methods and design of novel vaccine. Hopefully, these analyses will be useful for PRRS control strategy. Considering that the modern transportation in pork supply chains can easily spread the virus nationwide or even internationally, and meanwhile the wide use of attenuated PRRSV live vaccine will raise the risk of reversion to virulence and increase the possibility of recombination between vaccine strains and field strains, the RPRSV diversity will be continually expanded and the epidemic situation in the field will be more and more complicated. So if we try to gain a deeper view of the PRRSV epidemiology, the long-term investigation, linked observation between genetic diversity and phenotypic difference, and effort of explaining the mechanism of how HP-PRRSV strains gain the dominance in field should be first concerned in future.

## 5. Conclusion

Our analysis results indicated that the genetic diversity of PRRSV in the field further increased in recent years in China, due to the dramatic variations of NSP2 and ORF5 genes of PRRSV, and the 30-aa deletion in NSP2-coding region should be no longer defined as the only molecular marker of the Chinese HP-PRRSV as the PRRSV strain with same deletion and low pathogenicity emerged in the field and the attenuated live vaccines derived from HP-PRRSV were widely used in pig farms.

## Supplementary Material

The representative sequences of PRRSV strains used in this study were downloaded from GenBank and listed in Table S1. Meanwhile the similarity percentages of the deduced amino acids of nonstructural and structural proteins of four isolates (BJ1102, GZ1101, LN1101 and SD0901) compared with other Chinese representative strains were summarized in Table S2-S5.

## Figures and Tables

**Table 1:** Primers used for amplification and sequencing of PRRSV genome and NSP2 HV region and ORF3 and ORF5 genes.

Name	Primer sequence	Location*	Length (bp)
5′-Outer-R	TTTCACTTCATCCCCACCAC	800	819
5′-Inner-R	CCCCGTTCATAAACTTGTAGAG	542	563
W1F	ATGACGTATAGGTGTTGGCTCT	1	
W1R	TACTCTTTCAGGAAGGGTGG	1575	1575
W2F	ACGCTCTGGTGCGACTACTA	1362	
W2R	AGGTTGTTCGGTTGTCTGATT	2253	892
W3F	CCTCCGTGGCGCAACAAGTCTTG	2115	
W3R	CGATGATGGCTTGAGCTGAGTAT	3178	1064
W4F	TGAGCCTCTGGATTTGTCTGC	2949	
W4R	GGCGATCTCATTAGGAGCAGTT	4329	1381
W5F	TGCTTAGGCTTGGCATTGTTG	4214	
W5R	ACGGTGTTCAGTGAGGGCTTT	5564	1351
W6F	ACTAACATTGCTGGTCTCGTCA	5350	
W6R	AAGGAAATCCAAGTCCTCGTC	6750	1401
W7F	TTGTGACCTCGCCAGTCCCAGTG	6500	
W7R	CCAAAGCGTGCCATCAATCCC	7922	1423
W8F	GGTTGATGGTGGTGTTGTGCT	7787	
W8R	GTCTTCTTTGGGTCCGTCTGG	9226	1440
W9F	TGGTCACCCTCATGGCCTTCT	9038	
W9R	CAAATACATAGCAATGGGAGTCAAA	10323	1286
W10F	TTCCTGGATGAAGCGGCGTAT	10194	
W10R	AACTCGGATGTATGAGGCGTAG	11573	1380
W11F	GGTGCTGGAAAGTGATGTTGG	11440	
W11R	AAAGCGGGCATACCGTGTAAT	12774	1335
W12F	AGTGGTTTGGATGTGGTGGCT	12400	
W12R	TGTTGTTGTTGCTGGCGTTGA	13803	1404
W13F	ATGTGCGACTGCTTCATTTCA	13599	
W13R	TTTGCTGCTTGCCGTTGTTAT	14826	1228
W14F	TCCACTACGGTCAACGGCACAT	14702	
W14R	GGATCCGGTACCTCTAGATCAGA		637
3′race adaptor-T	GGATCCGGTACCTCTAGATCAGATTTTTTTTTTTTTTTTTT		
Nsp2-F	CCTCCGTGGTGCAACAAATCTTG	2115	
Nsp2-R	CGATGATGGCTTGAGCTGAGTAT	3178	1064/1154
ORF3-F	CAGGGTCAAATGTAACCATAGTG	12506	
ORF3-R	GGCAAGAAGAAAGCATGAGGAG	13457	952
ORF5-F	AGCCTGTCTTTTTGCCATTCT	13654	
ORF5-R	CTTTTGTGGAGCCGTGCTATC	14335	682
ORF7F	TGATAACCACGCATTTGTCG	14668	
ORF7R	GCCATTCACCACACATTCTTC	15228	561

*The location is according to the genomic sequences of JXwn06 (GenBank Accession number: EF641008).

**Table 2:** Geographic origin and amplified sequence size from clinical samples in this study.

Serial number	Sample designation*	Collection date	Area	NSP2 ( bp )	ORF3 ( bp )	ORF5 ( bp )	Serial number	Sample designation	Collection date	Area	NSP2 ( bp )	ORF3 ( bp )	ORF5 ( bp )
1	AHQS0901	2009.08	Anhui	1014	765	603	65	HB0908	2009.12	Hebei	1014	765	NA
2	AHQS0902	2009.08	Anhui	NA^#^	765	603	66	HB0909	2009.12	Hebei	1014	765	603
3	AHQS0903	2009.08	Anhui	1014	765	603	67	HB1001	2010.04	Hebei	1014	765	603
4	AHQS0905	2009.08	Anhui	1014	765	603	68	HB1002	2010.04	Hebei	1014	765	603
5	AHQS0906	2009.08	Anhui	NA	765	603	69	HB1101	2011.01	Hebei	1104	765	603
6	AHQS0907	2009.08	Anhui	1014	765	603	70	HB1102	2011.01	Hebei	1014	765	603
7	AHQS0908	2009.08	Anhui	NA	765	603	71	HB1103	2011.11	Hebei	1104	765	603
8	AHQS0909	2009.08	Anhui	NA	765	603	72	HB1104	2011.11	Hebei	1014	765	603
9	AHQS0910	2009.08	Anhui	NA	765	603	73	HB1105	2011.03	Hebei	1014	765	603
10	AHSS0901	2009.08	Anhui	1014	765	603	74	HB1106	2011.04	Hebei	1014	765	NA
11	AHSS0902	2009.08	Anhui	1014	765	603	75	HB1201	2012.05	Hebei	1014	765	603
12	AHSS0903	2009.08	Anhui	1014	765	603	76	HEN1001	2010.03	Henan	NA	NA	603
13	AHSS0904	2009.08	Anhui	NA	765	603	77	HEN1002	2010.11	Henan	1014	765	603
14	AHSS0905	2009.08	Anhui	NA	765	603	78	HEN1003	2010.11	Henan	1014	765	603
15	BJ0803	2008.04	Beijing	1014	765	603	79	HEN1101	2011.01	Henan	1014	765	603
16	BJ0804	2008.09	Beijing	1014	765	603	80	HEN1102	2011.09	Henan	1104	765	603
17	BJ0805	2008.09	Beijing	1014	765	603	81	HEN1103	2011.09	Henan	1014	765	603
18	BJ0806	2008.01	Beijing	1014	765	603	82	HLJ1101	2011.09	Heilongjiang	1014	765	603
19	BJ0807	2008.01	Beijing	1014	765	603	83	HLJ1102	2011.09	Heilongjiang	1014	765	603
20	BJ0808	2008.01	Beijing	NA	765	603	84	HLJ1103	2011.09	Heilongjiang	1014	765	603
21	BJ0809	2008.01	Beijing	NA	765	603	85	HLJ1104	2011.09	Heilongjiang	1014	765	603
22	BJ0810	2008.11	Beijing	1104	765	603	86	HN0901	2009.03	Henan	1014	765	NA
23	BJ0901	2009.02	Beijing	1014	765	603	87	HN0902	2009.05	Henan	1014	765	603
24	BJ0902	2009.02	Beijing	1014	765	603	88	HN0903	2009.09	Henan	1104	765	603
25	BJ0903	2009.04	Beijing	NA	765	603	89	HuB1001	2010.08	Hubei	1014	765	603
26	BJ0904	2009.05	Beijing	1014	NA	NA	90	HuN1001	2010.08	Hunan	1104	765	NA
27	BJ0905	2009.05	Beijing	1104	765	603	91	HuN1002	2010.12	Hunan	1014	765	603
28	BJ0906	2009.05	Beijing	NA	765	603	92	HuN1003	2010.12	Hunan	1014	765	603
29	BJ0907	2009.05	Beijing	NA	NA	603	93	HuN1101	2011.11	Hunan	1014	765	603
30	BJ0908	2009.06	Beijing	1014	765	603	94	HuN1102	2011.01	Hunan	1014	NA	603
31	BJ0909	2009.09	Beijing	1104	765	603	95	JL1101	2011.01	Jilin	1104	765	603
32	BJ0910	2009.11	Beijing	NA	765	603	96	JL1102	2011.03	Jilin	1014	765	603
33	BJ0911	2009.12	Beijing	NA	765	603	97	JX1001	2010.08	Jiangxi	1014	765	603
34	BJ0912	2009.12	Beijing	NA	765	603	98	JX1002	2010.09	Jiangxi	1014	765	603
35	BJ1001	2010.03	Beijing	1014	765	603	99	LN0801	2008.01	Liaoning	1014	765	603
36	BJ1002	2010.03	Beijing	1104	765	603	100	LN0901	2009.06	Liaoning	1014	765	603
37	BJ1003	2010.05	Beijing	1014	765	603	101	LN0902	2009.11	Liaoning	1014	765	603
38	BJ1004	2010.07	Beijing	1014	765	603	102	LN1001	2010.09	Liaoning	1014	765	603
39	BJ1005	2010.07	Beijing	1014	765	603	103	LN1101	2011.01	Liaoning	1050	765	603
40	BJ1006	2010.08	Beijing	1104	765	603	104	NMG0801	2008.09	Inner Mongolia	NA	765	NA
41	BJ1007	2010.09	Beijing	1014	765	603	105	NMG0802	2008.09	Inner Mongolia	NA	765	NA
42	BJ1008	2010.09	Beijing	1014	765	603	106	SC0801	2008.01	Sichuan	1014	765	603
43	BJ1009	2010.11	Beijing	NA	765	NA	107	SC1101	2011.03	Sichuan	1014	765	NA
44	BJ1010	2010.11	Beijing	1014	765	603	108	SC1201	2012.04	Sichuan	1014	765	603
45	BJ1101	2011.01	Beijing	1014	765	603	109	SD0901	2009.04	Shandong	1014	765	603
46	BJ1102	2011.01	Beijing	1014	765	603	110	SD0902	2009.12	Shandong	1014	765	603
47	GX1001	2010.06	Guangxi	1014	765	603	111	SD0903	2009.12	Shandong	1014	765	603
48	GX1002	2010.06	Guangxi	1014	765	603	112	SD0904	2009.12	Shandong	1014	765	603
49	GZ1001	2010.09	Guizhou	1014	765	603	113	SD0905	2009.12	Shandong	1014	765	603
50	GZ1002	2010.11	Guizhou	1014	765	603	114	SD0906	2009.12	Shandong	1014	765	603
51	GZ1101	2011.03	Guizhou	1095	765	600	115	SD1001	2010.03	Shandong	1014	765	603
52	HB0801	2008.07	Hebei	NA	765	603	116	SD1002	2010.03	Shandong	NA	765	603
53	HB0802	2008.01	Hebei	1014	765	603	117	SD1003	2010.11	Shandong	1014	765	603
54	HB0803	2008.01	Hebei	1014	765	603	118	SD1004	2010.11	Shandong	1014	765	603
55	HB0804	2008.01	Hebei	NA	765	603	119	SD1101	2011.03	Shandong	1014	765	603
56	HB0805	2008.01	Hebei	NA	NA	603	120	SX1101	2011.05	Shanxi	1014	765	603
57	HB0806	2008.11	Hebei	1014	765	603	121	TJ0901	2009.11	Tianjin	NA	765	603
58	HB0901	2009.03	Hebei	NA	765	NA	122	TJ0902	2009.11	Tianjin	NA	765	603
59	HB0902	2009.03	Hebei	1014	765	603	123	TJ0903	2009.11	Tianjin	NA	765	603
60	HB0903	2009.03	Hebei	1014	765	603	124	TJ1101	2011.04	Tianjin	1014	765	603
61	HB0904	2009.04	Hebei	1014	765	603	125	ZJ1001	2010.01	Zhejiang	1014	765	603
62	HB0905	2009.11	Hebei	1104	765	603	126	ZJ1101	2011.02	Zhejiang	1014	765	603
63	HB0906	2009.11	Hebei	1104	765	603	127	ZJ1102	2011.04	Zhejiang	1014	765	603
64	HB0907	2009.11	Hebei	1014	765	603	128	ZJ1103	2011.04	Zhejiang	1014	765	603

*Each sample was named according to the region and collection year; ^#^NA: not amplified.
