# Supplementary material for: Genetic Diversity Analysis of Genotype 2 Porcine Reproductive and Respiratory Syndrome Viruses Emerging in Recent Years in China
Source: Biomed Res Int. 2014 Feb 25;2014:748068. doi: 10.1155/2014/748068 (PMC3955690; doi:10.1155/2014/748068)
Supplement: Supplementary file 1 — The representative sequences of PRRSV strains used in this study were downloaded from GenBank and listed in Table S1. Meanwhile the similarity percentages of the deduced amino acids of nonstructural and structural proteins of four isolates (BJ1102, GZ1101, LN1101 and SD0901) compared with other Chinese representative strains were summarized in Table S2-S5. [file 748068.f1.docx]

**Table S1** PRRSV referenced strains used in this study

| Name | GenBank accession no. | Origin | Year of isolation | Name | GenBank accession no. | Origin | Year of isolation |
| --- | --- | --- | --- | --- | --- | --- | --- |
| Lelystad virus* | M96262 | Netherlands | 1993 | SY0608 | EU144079 | China | 2007 |
| VR-2332 | U87392 | United States | 1993 | WUH1 | EU187484 | China | 2007 |
| BJ-4 | AF331831 | China | 1996 | 08HuN | GU169411 | China | 2008 |
| CH-1a | AY032626 | China | 1996 | CWZ-1-F3 | FJ889130 | China | 2008 |
| PL97-1 | AY585241 | Korea | 1997 | GDBY1 | GQ374442 | China | 2008 |
| 16244B | AF046869 | United States | 1998 | NT0801 | HQ315836 | China | 2008 |
| NVSL 97-7985 IA 1-4-2 | AF325691 | United States | 2000 | YN2008 | EU880435 | China | 2008 |
| HB-1(sh)/2002 | AY150312 | China | 2002 | YN9 | GU232738 | China | 2008 |
| HB-2(sh)/2002 | AY262352 | China | 2002 | 09HEB | JF268679 | China | 2009 |
| P129 | AF494042 | United States | 2002 | 09HEN1 | JF268684 | China | 2009 |
| GS2003 | EU880442 | China | 2003 | 09HUB1 | JF268682 | China | 2009 |
| HN1 | AY457635 | China | 2003 | SD0901 | JN256115 | China | 2009 |
| JA142 | AY424271 | United States | 2003 | SX2009 | FJ895329 | China | 2009 |
| NB/04 | FJ536165 | China | 2004 | YD | JF748717 | China | 2009 |
| MN184A | DQ176019 | United States | 2005 | 10-10QN | JQ663556 | China | 2010 |
| SHB | EU864232 | China | 2005 | 10-LW3-7 | JQ663564 | China | 2010 |
| BJsy06 | EU097707 | China | 2006 | DC | JF748718 | China | 2010 |
| CC-1 | EF153486 | China | 2006 | FS | JF796180 | China | 2010 |
| HEB1 | EF112447 | China | 2006 | GX1003 | JX912249 | China | 2010 |
| HUB2 | EF112446 | China | 2006 | QY2010 | JQ743666 | China | 2010 |
| Jsyx | EU939312 | China | 2006 | DY | JN864948 | China | 2011 |
| JX143 | EU708726 | China | 2006 | GM2 | JN662424 | China | 2011 |
| JXA1 | EF112445 | China | 2006 | HH08 | JX679179 | China | 2011 |
| JXwn06 | EF641008 | China | 2006 | NVDC-GD2-2011 | JQ715697 | China | 2011 |
| TJ | EU860248 | China | 2006 | QYYZ | JQ308798 | China | 2011 |
| TP | EU864233 | China | 2006 | YN-2011 | JX857698 | China | 2011 |
| BJ0706 | GQ351601 | China | 2007 | 10-10JL | JQ663554 | China | 2012 |
| CG | EU864231 | China | 2007 | JL-04/12 | JX177644 | China | 2012 |
| Em2007 | EU262603 | China | 2007 | SD16 | JX087437 | China | 2012 |
| GD | EU825724 | China | 2007 | CH-1R | EU807840 | China | Vaccine |
| GDQJ | GQ374441 | China | 2007 | Ingelvac ATP | DQ988080 | United States | Vaccine |
| GDQY1 | JN387271 | China | 2007 | JXA1 P80 | FJ548853 | China | Vaccine |
| Henan-1 | EU200962 | China | 2007 | Prime Pac | DQ779791 | United States | Vaccine |
| HuN4 | EF635006 | China | 2007 | RespPRRS MLV | AF066183 | United States | Vaccine |
| Shaanxi-2 | HQ401282 | China | 2007 | SP | AF184212 | Singapore | Vaccine |

* Genotype 1 PRRSV strain

**Table S2** Similarity analysis on the deduced amino acids of nonstructural and structural proteins of BJ1102 with those of VR-2332, BJ-4, CH-1a, NB04, HB-1(sh)/2002, HB-2(sh)/2002, HuN4, JXwn06, JXA1, JXA1 P80 (%)

| ORF | Cleavage Products | VR-2332 | BJ-4 | CH-1a | NB04 | HB-1(sh)/2002 | HB-2(sh)/2002 | HuN4 | JXwn06 | JXA1 | JXA1 P80 |
| --- | --- | --- | --- | --- | --- | --- | --- | --- | --- | --- | --- |
| Nonstructural proteins | | | | | | | | | | | |
| 1a | NSP1α | 95.8 | 95.8 | 97.0 | 99.4 | 98.2 | 98.2 | 100.0 | 100.0 | 100.0 | 100.0 |
|  | NSP1β | 83.6 | 82.6 | 86.8 | 95.4 | 95.4 | 83.1 | 97.7 | 97.3 | 97.3 | 96.8 |
|  | NSP2 | 76.3 | 75.9 | 86.2 | 92.5 | 91.4 | 84.3 | 94..4 | 94.8 | 94.4 | 94.1 |
|  | NSP3 | 93.7 | 93.7 | 97.3 | 98.2 | 97.1 | 96.6 | 98.4 | 98.9 | 98.4 | 98.2 |
|  | NSP4 | 94.6 | 94.6 | 96.6 | 99.0 | 99.0 | 96.1 | 100.0 | 100.0 | 100.0 | 99.5 |
|  | NSP5 | 92.4 | 93.0 | 94.2 | 97.7 | 97.7 | 93.6 | 98.8 | 98.8 | 98.8 | 98.8 |
|  | NSP6 | 94.1 | 94.1 | 100.0 | 100.0 | 100.0 | 100.0 | 100.0 | 100.0 | 100.0 | 100.0 |
|  | NSP7 | 89.6 | 88.5 | 96.5 | 96.5 | 96.9 | 95.8 | 99.6 | 99.2 | 99.6 | 99.2 |
|  | NSP8 | 100.0 | 100.0 | 100.0 | 100.0 | 97.9 | 97.9 | 100.0 | 100.0 | 100.0 | 100.0 |
| 1b | NSP9 | 98.0 | 97.7 | 99.1 | 99.7 | 99.4 | 98.6 | 99.5 | 100.0 | 99.7 | 99.8 |
|  | NSP10 | 95.9 | 95.9 | 97.5 | 99.5 | 98.4 | 97.1 | 99.5 | 99.5 | 98.9 | 99.1 |
|  | NSP11 | 94.6 | 95.5 | 98.2 | 98.7 | 98.2 | 96.9 | 99.6 | 99.6 | 99.6 | 99.6 |
|  | NSP12 | 94.8 | 94.8 | 96.8 | 97.4 | 96.8 | 96.1 | 99.4 | 99.4 | 99.4 | 99.4 |
| Structural proteins | | | | | | | | | | | |
| ORF2a | GP2a | 93.8 | 93.0 | 95.7 | 96.9 | 95.7 | 93.0 | 98.4 | 98.8 | 98.8 | 96.5 |
| ORF2b | ORF2b | 89.3 | 88.0 | 93.3 | 96.0 | 93.3 | 93.2 | 98.7 | 98.7 | 98.7 | 96.0 |
| ORF3 | GP3 | 85.2 | 85.9 | 92.6 | 94.5 | 92.6 | 90.6 | 97.7 | 98.0 | 97.3 | 97.7 |
| ORF4 | GP4 | 90.6 | 90.6 | 98.9 | 98.3 | 98.9 | 96.6 | 98.3 | 98.3 | 96.1 | 98.9 |
| ORF5 | GP5 | 88.5 | 86.6 | 91.6 | 94.1 | 91.6 | 89.6 | 97.0 | 97.0 | 97.0 | 95.5 |
| ORF6 | M | 97.2 | 97.2 | 97.7 | 98.9 | 99.4 | 98.3 | 100.0 | 100.0 | 100.0 | 99.4 |
| ORF7 | N | 96.0 | 96.0 | 94.4 | 97.6 | 92.7 | 97.6 | 99.2 | 99.2 | 99.2 | 99.2 |

**Table S3** Similarity analysis on the deduced amino acids of nonstructural and structural proteins of GZ1101 with those of VR-2332, BJ-4, CH-1a, NB04, HB-1(sh)/2002, HB-2(sh)/2002, HuN4, JXwn06, JXA1, JXA1 P80 (%)

| ORF | Cleavage Products | VR-2332 | BJ-4 | CH-1a | NB04 | HB-1(sh)/2002 | HB-2(sh)/2002 | HuN4 | JXwn06 | JXA1 | JXA1 P80 |
| --- | --- | --- | --- | --- | --- | --- | --- | --- | --- | --- | --- |
| Nonstructural proteins | | | | | | | | | | | |
| 1a | NSP1α | 99.4 | 99.4 | 99.4 | 95.8 | 94.5 | 97.6 | 96.4 | 96.4 | 96.4 | 96.4 |
|  | NSP1β | 93.2 | 93.2 | 82.6 | 81.7 | 82.6 | 81.3 | 82.2 | 81.7 | 81.7 | 81.7 |
|  | NSP2 | 96.6 | 96.2 | 80.5 | 77.1 | 76.9 | 77.3 | 76.6 | 76.9 | 76.5 | 76.5 |
|  | NSP3 | 98.7 | 98.7 | 96.0 | 94.9 | 94.2 | 95.7 | 94.9 | 95.3 | 94.9 | 94.6 |
|  | NSP4 | 99.5 | 99.5 | 97.1 | 95.6 | 95.6 | 96.6 | 95.1 | 95.1 | 95.1 | 94.6 |
|  | NSP5 | 97.1 | 97.1 | 93.0 | 93.6 | 92.4 | 93.6 | 93.0 | 93.0 | 93.0 | 93.0 |
|  | NSP6 | 100.0 | 100.0 | 94.1 | 94.1 | 94.1 | 94.1 | 94.1 | 94.1 | 94.1 | 94.1 |
|  | NSP7 | 98.1 | 96.9 | 91.5 | 90.0 | 90.8 | 90.0 | 89.6 | 89.2 | 89.6 | 89.2 |
|  | NSP8 | 97.9 | 97.9 | 97.9 | 97.9 | 95.7 | 95.7 | 97.9 | 97.9 | 97.9 | 97.9 |
| 1b | NSP9 | 99.4 | 99.1 | 97.4 | 97.4 | 97.7 | 97.2 | 97.2 | 97.7 | 97.4 | 97.5 |
|  | NSP10 | 99.5 | 99.8 | 95.9 | 96.6 | 95.9 | 96.2 | 96.6 | 96.6 | 95.9 | 96.2 |
|  | NSP11 | 97.3 | 99.1 | 94.6 | 94.2 | 94.2 | 94.2 | 94.6 | 95.1 | 94.6 | 94.2 |
|  | NSP12 | 98.7 | 98.7 | 95.5 | 96.1 | 95.5 | 94.8 | 95.5 | 95.5 | 95.5 | 95.5 |
| Structural proteins | | | | | | | | | | | |
| ORF2a | GP2a | 98.8 | 98.1 | 95.0 | 92.6 | 93.8 | 91.4 | 92.2 | 92.6 | 92.6 | 91.9 |
| ORF2b | ORF2b | 98.7 | 97.3 | 92.0 | 94.7 | 92.0 | 94.6 | 92.0 | 92.0 | 92.0 | 92.0 |
| ORF3 | GP3 | 94.9 | 95.3 | 89.1 | 88.3 | 89.1 | 88.6 | 86.3 | 86.3 | 85.9 | 87.1 |
| ORF4 | GP4 | 97.8 | 97.8 | 91.7 | 90.0 | 91.7 | 89.9 | 91.7 | 91.7 | 89.4 | 91.7 |
| ORF5 | GP5 | 95.5 | 95.0 | 90.0 | 86.6 | 87.6 | 88.1 | 87.6 | 87.6 | 87.1 | 86.6 |
| ORF6 | M | 98.9 | 98.9 | 98.3 | 98.3 | 98.9 | 98.9 | 98.3 | 98.3 | 98.3 | 97.7 |
| ORF7 | N | 100.0 | 96.8 | 100.0 | 96.8 | 95.2 | 96.8 | 95.2 | 95.2 | 95.2 | 95.2 |

**Table S4** Similarity analysis on the deduced amino acids of nonstructural and structural proteins of LN1101 with those of VR-2332, BJ-4, CH-1a, NB04, HB-1(sh)/2002, HB-2(sh)/2002, HuN4, JXwn06, JXA1, JXA1 P80 (%)

| ORF | Cleavage Products | VR-2332 | BJ-4 | CH-1a | NB04 | HB-1(sh)/2002 | HB-2(sh)/2002 | HuN4 | JXwn06 | JXA1 | JXA1 P80 |
| --- | --- | --- | --- | --- | --- | --- | --- | --- | --- | --- | --- |
| Nonstructural proteins | | | | | | | | | | | |
| 1a | NSP1α | 96.4 | 96.4 | 96.4 | 98.8 | 97.6 | 97.6 | 99.4 | 99.4 | 99.4 | 99.4 |
|  | NSP1β | 86.3 | 85.4 | 89.5 | 96.3 | 97.7 | 86.3 | 95.4 | 95.0 | 95.0 | 95.4 |
|  | NSP2 | 78.5 | 78.1 | 89.0 | 95.6 | 97.6 | 84.8 | 93.6 | 93.9 | 93.6 | 93.0 |
|  | NSP3 | 94.6 | 94.6 | 98.0 | 98.7 | 99.1 | 97.3 | 98.4 | 98.9 | 98.4 | 98.2 |
|  | NSP4 | 95.1 | 95.1 | 97.6 | 100.0 | 100.0 | 96.1 | 99.0 | 99.0 | 99.0 | 98.5 |
|  | NSP5 | 92.4 | 93.0 | 95.9 | 99.4 | 99.4 | 93.6 | 99.4 | 99.4 | 99.4 | 99.4 |
|  | NSP6 | 94.1 | 94.1 | 100.0 | 100.0 | 100.0 | 100.0 | 100.0 | 100.0 | 100.0 | 100.0 |
|  | NSP7 | 90.0 | 89.6 | 91.5 | 90.0 | 90.8 | 90.0 | 89.6 | 89.2 | 89.6 | 89.2 |
|  | NSP8 | 100.0 | 100.0 | 100.0 | 100.0 | 97.9 | 97.9 | 100.0 | 100.0 | 100.0 | 100.0 |
| 1b | NSP9 | 98.1 | 97.8 | 98.9 | 99.2 | 99.8 | 98.8 | 99.1 | 99.5 | 99.2 | 99.4 |
|  | NSP10 | 95.0 | 95.0 | 97.1 | 98.2 | 98.9 | 96.6 | 98.2 | 98.2 | 97.5 | 97.7 |
|  | NSP11 | 94.2 | 95.1 | 97.8 | 99.1 | 98.7 | 96.4 | 98.2 | 98.2 | 98.2 | 98.2 |
|  | NSP12 | 96.1 | 96.1 | 98.1 | 98.7 | 98.1 | 97.4 | 98.1 | 98.1 | 98.1 | 98.1 |
| Structural proteins | | | | | | | | | | | |
| ORF2a | GP2a | 93.4 | 92.6 | 97.3 | 98.8 | 97.7 | 94.6 | 97.7 | 98.1 | 98.1 | 96.1 |
| ORF2b | ORF2b | 93.3 | 92.0 | 97.3 | 100.0 | 97.3 | 97.3 | 97.3 | 97.3 | 97.3 | 97.3 |
| ORF3 | GP3 | 89.1 | 89.8 | 94.9 | 96.5 | 94.9 | 92.9 | 95.7 | 95.3 | 95.3 | 95.3 |
| ORF4 | GP4 | 89.4 | 90.0 | 96.7 | 96.7 | 96.7 | 93.3 | 96.1 | 95.6 | 93.9 | 96.6 |
| ORF5 | GP5 | 88.6 | 87.1 | 93.1 | 94.6 | 94.6 | 91.1 | 94.6 | 94.6 | 94.6 | 93.6 |
| ORF6 | M | 97.2 | 97.2 | 97.7 | 98.9 | 99.4 | 98.3 | 98.9 | 98.9 | 98.9 | 98.3 |
| ORF7 | N | 96.8 | 96.8 | 95.2 | 98.4 | 92.7 | 100.0 | 96.8 | 96.8 | 96.8 | 96.8 |

**Table S5** Similarity analysis on the deduced amino acids of nonstructural and structural proteins of SD0901 with those of VR-2332, BJ-4, CH-1a, NB04, HB-1(sh)/2002, HB-2(sh)/2002, HuN4, JXwn06, JXA1, JXA1 P80 (%)

| ORF | Cleavage Products | VR-2332 | BJ-4 | CH-1a | NB04 | HB-1(sh)/2002 | HB-2(sh)/2002 | HuN4 | JXwn06 | JXA1 | JXA1 P80 |
| --- | --- | --- | --- | --- | --- | --- | --- | --- | --- | --- | --- |
| Nonstructural proteins | | | | | | | | | | | |
| 1a | NSP1α | 95.8 | 95.8 | 97.0 | 99.4 | 98.2 | 98.2 | 100.0 | 100.0 | 100.0 | 100.0 |
|  | NSP1β | 85.8 | 84.9 | 88.1 | 97.3 | 97.3 | 84.5 | 99.1 | 98.6 | 98.6 | 98.2 |
|  | NSP2 | 77.1 | 76.3 | 87.3 | 94.7 | 93.5 | 84.5 | 97.1 | 97.4 | 97.1 | 96.7 |
|  | NSP3 | 94.4 | 94.4 | 98.0 | 98.9 | 97.5 | 97.3 | 99.1 | 99.6 | 99.1 | 98.9 |
|  | NSP4 | 94.6 | 94.6 | 96．6 | 99.0 | 99.0 | 96.1 | 100.0 | 100.0 | 100.0 | 99.5 |
|  | NSP5 | 93.6 | 94.2 | 94.2 | 97.7 | 97.7 | 93.6 | 98.8 | 98.8 | 98.8 | 94.2 |
|  | NSP6 | 94.1 | 94.1 | 100.0 | 100.0 | 100.0 | 100.0 | 100.0 | 100.0 | 100.0 | 100.0 |
|  | NSP7 | 90.4 | 89.2 | 96.2 | 96.2 | 96.5 | 95.8 | 99.2 | 98.8 | 99.2 | 98.8 |
|  | NSP8 | 100.0 | 100.0 | 100.0 | 100.0 | 97.9 | 97.9 | 100.0 | 100.0 | 100.0 | 100.0 |
| 1b | NSP9 | 97.5 | 97.2 | 98.9 | 99.2 | 98.9 | 98.1 | 99.1 | 99.5 | 99.2 | 99.4 |
|  | NSP10 | 96.4 | 96.4 | 98.0 | 100.0 | 98.9 | 97.5 | 100.0 | 100.0 | 99.3 | 99.5 |
|  | NSP11 | 94.2 | 95.1 | 97.8 | 98.2 | 97.8 | 96.4 | 99.1 | 99.1 | 99.1 | 99.6 |
|  | NSP12 | 95.5 | 95.5 | 97.4 | 98.1 | 97.4 | 96.8 | 100.0 | 100.0 | 100/0 | 100.0 |
| Structural proteins | | | | | | | | | | | |
| ORF2a | GP2a | 93.0 | 92.2 | 96.1 | 97.3 | 96.1 | 93.4 | 99.2 | 99.6 | 99.6 | 97.3 |
| ORF2b | ORF2b | 90.7 | 89.3 | 94.7 | 97.3 | 94.7 | 94.6 | 100.0 | 100.0 | 100.0 | 97.3 |
| ORF3 | GP3 | 85.5 | 86.3 | 93.4 | 95.7 | 93.4 | 91.4 | 98.8 | 99.2 | 98.4 | 98.0 |
| ORF4 | GP4 | 90.6 | 90.0 | 98.3 | 98.3 | 98.3 | 96.1 | 98.3 | 98.3 | 96.1 | 98.3 |
| ORF5 | GP5 | 88.6 | 86.6 | 93.6 | 97.0 | 94.6 | 91.6 | 100.0 | 100.0 | 99.5 | 98.0 |
| ORF6 | M | 96.6 | 96.6 | 97.2 | 98.3 | 98.9 | 97.7 | 99.4 | 99.4 | 99.4 | 98.9 |
| ORF7 | N | 95.2 | 95.2 | 95.2 | 98.4 | 91.9 | 86.8 | 100.0 | 100.0 | 100.0 | 100.0 |
